# Roadbook for the implementation of next-generation sequencing in clinical practice in oncology and hemato-oncology in Belgium

**DOI:** 10.1186/s13690-018-0295-z

**Published:** 2018-09-06

**Authors:** Els Van Valckenborgh, Aline Hébrant, Aline Antoniou, Wannes Van Hoof, Johan Van Bussel, Patrick Pauwels, Roberto Salgado, Waltruda Van Doren, Anouk Waeytens, Marc Van den Bulcke

**Affiliations:** 1 Belgian Cancer Centre, Sciensano, Brussels, Belgium; 2 Quality of Laboratories, Sciensano, Brussels, Belgium; 3 Healthdata.be, Sciensano, Brussels, Belgium; 4 Pathology Department, University Hospital of Antwerp, Antwerp, Belgium; 5 Department of Pathology, GZA Hospitals Antwerp, Antwerp, Belgium; 6 Division of Research, Peter Mac Callum Cancer Center, Melbourne, Australia; 7 Breast Cancer Translational Research Laboratory, Jules Bordet Institute, Brussels, Belgium; 8 Health Care Department, National Institute for Health and Disability Insurance, Brussels, Belgium

**Keywords:** Next-generation sequencing, Health care, Cancer, Personalized medicine

## Abstract

In the field of oncology research, next-generation sequencing has contributed significantly to the discovery of DNA mutations associated with diagnosis and prognosis. It also aids in the development of targeted therapies to specific mutations and the rise of personalized medicine. As part of molecular diagnostics in cancer patients, analysis by next-generation sequencing is becoming part of routine clinical practice. The introduction of this complex technology in a healthcare system comes with multiple challenges and requires a clear action plan. Such an action plan, as outlined in this paper, was developed in Belgium and includes steps in ensuring the quality and indications of NGS testing, installing data registration and tackling ethical issues. A final step is to perform a pilot study to control the access, quality, harmonization and expertise in DNA testing. This action plan can serve as a guide for similar initiatives by other countries to facilitate NGS implementation in clinical practice.

## Background

Personalized medicine, also known as individualized or precision medicine refers to “a medical model using the characteristics of individuals’ phenotypes and genotypes (e.g. molecular profiling, medical imaging, and lifestyle data) for tailoring the right therapeutic strategy for the right person at the right time” [1]. It is complementary to the ‘one size fits all’ medical approach wherein a single treatment strategy would fit all patients from a certain tumor type. One of the hallmarks of personalized medicine is the development of targeted agents. These targeted agents are designed to specifically interfere with key molecular events that are involved in the proliferation, survival or spread of cancer cells and aim harming as little as possible normal cell function. Potential targets are molecules overexpressed or mutated in tumor cells or fusion proteins that are newly formed by chromosomal rearrangements. Monoclonal antibodies and small molecules are the main types of drugs applied in targeted therapies. Examples are the BCR-ABL1 fusion leading to a constitutively active tyrosine kinase activity in chronic myeloid leukemia targetable by imatinib or the use of vemurafenib in melanoma patients targeting the *BRAFV600E* mutation [2, 3].

The concept of targeted therapy is driven by molecular diagnostics that enables the identification of patient populations most likely to benefit from precision medicine. Molecular diagnostics in oncology comprises a set of by now standard techniques to analyze cellular biomarkers including immunohistochemistry, immune-phenotyping, cytogenetics, polymerase chain reaction, fluorescent in situ hybridization, comparative genomic hybridization assays, microarrays and Sanger sequencing. Recent technical advances and lower cost have paved the introduction of next-generation sequencing (NGS) analysis in clinical routine practice. This high-throughput approach wherein millions of sequencing reads are analyzed in parallel allows investigating a large number of genes and samples simultaneously. Over the past years the cost of NGS tests has dropped significantly making it more affordable for reimbursement by the healthcare systems. The cost drivers of this technology are however shifting from the purely analytical phase towards the post-analytical phase such as data-storage, analysis and interpretation. NGS enables more precise decision-making for cancer patients by providing detailed information for diagnosis, prognosis and therapy with targeted drugs or conventional treatment schedules (chemotherapeutics, stem cell transplantation). In order to achieve this precision-decision making, a strict quality assurance and data registration to allow downstream data-mining and outcome analysis of stratified medicine usage is required. NGS can be performed on tumor tissue (formalin-fixed, paraffin-embedded (FFPE) samples) as well as on liquid samples (blood, bone marrow), therefore, allowing the technology to be implemented both in oncology and hemato-oncology [4, 5]. Introducing a complex new technology in a healthcare system aiming at offering full access to all citizens represents a major challenge which requires ahead planning.

## Main text

**Fig. 1:**
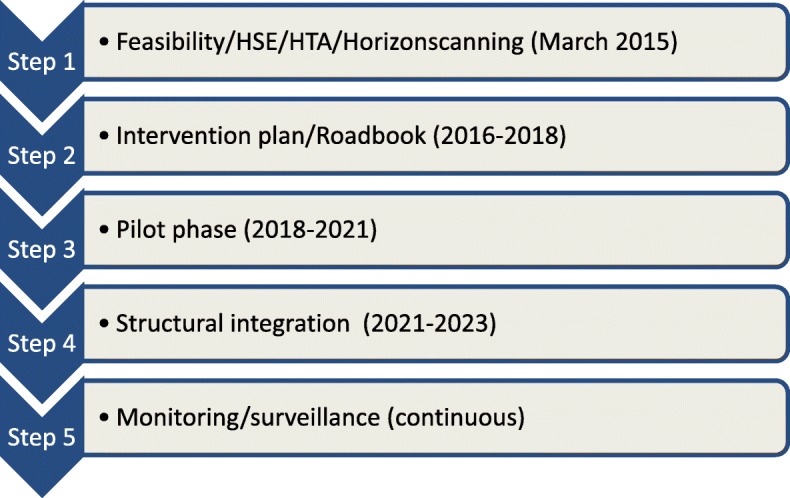
Introduction of Next-Generation-Sequencing in routine diagnostics in oncology and hemato-oncology in Belgium: a multistep process. HSE: Health Service Evaluation; HTA: Health Technology Assessment

A step-wise process has been applied in Belgium wherein, at first, feasibility was assessed on the use of NGS in routine clinical diagnostics in oncology and hemato-oncology [6] (Fig. 1). The conclusions of this assessment were discussed with and endorsed in 2015 by the major stakeholders in Belgium, including representatives of public bodies active in healthcare, patient associations and health insurance funds. In a second step, the conclusions of the feasibility study were translated into a roadbook that describes the major actions to be taken to introduce NGS in clinical settings. This roadbook entitled “Introduction of Next-Generation Sequencing in routine diagnostics in oncology and hemato-oncology in Belgium” was officially approved by the Ministry of Social Affairs and Health in 2016 (see Table 1). The roadbook includes 1°) a description of the governance structure of the intervention, 2°) the major technical and logistic actions to be undertaken including an allocated budget, and 3°) a number of awareness raising initiatives for health professionals and patient/citizens.

**Table 1:** Overview of the actions for the stepwise introduction of NGS technology in oncology and hemato-oncology: the Belgian strategy

Actions of the Belgian roadbook for the implementation of next-generation sequencing in clinical practice in oncology and hemato-oncology	
ACTION 1	Establish a commission: Commission of Personalized Medecine (ComPerMed)
ACTION 2	Development of guidelines for NGS use
ACTION 3	Development of criteria for NGS use
ACTION 4 & 5	Develop and organize a benchmarking trial and EQA for NGS use
ACTION 6	Implement NGS registration, storage and data management
ACTION 7	Provide NGS education and training
ACTION 8	Informed consent, legal and ethical implications of NGS use molecular diagnostics
ACTION 9	Pilot study ‘NGS use in routine diagnostics’
ACTION 10	Build on hospital networks for NGS use

### Action 1: Establish a commission: Commission of Personalized Medicine

**Fig. 2:**
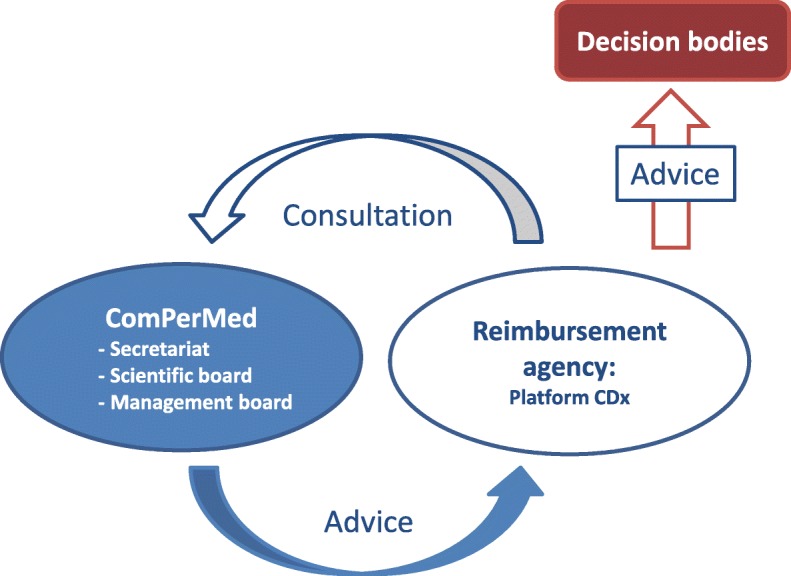
Schematic representation of the ComPerMed and its activities as advisory board towards the reimbursement agency specifically the Platform CDx. This Platform is a mixed working group of the CRM/TMC, Commission for Reimbursement of Medicines/Technical Medical Council, the decision bodies for medicines and diagnostic tests, respectively

The effective implementation of NGS and, in a broader context, also personalized medicine in healthcare, requires support by a multidisciplinary committee of experts, ideally embedded in an international framework. Such committee was created end 2016 and designated as the ‘Commission of Personalized Medicine’ or ‘ComPerMed’. The mission of the ComPerMed is to provide, as much as possible, evidence-based advice to policymakers on the relevance of introducing innovative solutions in personalized medicine, with a major focus on ‘omics’ technologies. The ComPerMed works closely together with another novel advisory body, the so-called Platform CDx of the NIHDI. The Platform CDx gathers competences of two advisory bodies in the NIHDI: the Commission for Reimbursement of Medicines and the Technical Medical Council (Fig. 2). While the latter provides advice on the practices and the tests to be reimbursed by the healthcare system, the former advices on the approval of the medicines. In the last two years, the ComPerMed developed guidelines on the technical requirements of NGS testing and the clinical utility of somatic mutations in cancer patients, and developed clinical diagnostic guidelines. The ComPerMed will also in the future assess the conditions for implementing other novel ‘omics‘technologies in daily oncology practice such as whole-genome sequencing (WGS), proteomics, metabolomics.

The ComPerMed is composed of a management board and a scientific board. The scope of activities performed by the ComPerMed are decided yearly by the management board which includes representatives of all concerned parties and decision bodies in this field: the sickness insurance agency (NIHDI), the Federal Ministry of Public Health, food chain safety and environment, the College of oncology, the College of genetics, the Commission of anatomic pathology, the Commission Clinical Biology, the Cancer Registry, the Federal agency of medicines and health products, the test accreditation organization (Belac), the Belgian Health Care Knowledge Centre (KCE) and Sciensano. They define the action plan of the ComPerMed and validate the final outcomes. The scientific board on the other hand is a broad representation of the different professionals and experts in the field of personalized medicine in Belgium, more specifically, clinicians, pathologists, clinical biologists, molecular biologists, bioinformatics and IT specialists. The scientific board works on a project basis with ad hoc working groups with experts in the topic of interest. The ComPerMed is mainly supporting action 2 and 3 of the Roadbook but can also be implicated in other actions when its members’ expertise is relevant. The Cancer Centre of Sciensano is in charge of the secretariat of the ComPerMed.

### Action 2: Development of guidelines for NGS use in oncology and hemato-oncology

Performing high quality diagnostic analysis within a healthcare system using complex tools such as NGS, both at the technical level as on the level of data interpretation, requires an overall agreement between the health professionals on practices. Guidelines represent a most valuable asset in such respect. Indeed, the NGS technology is complicated demanding both optimal assay conditions and settings in the “wet bench” part and “dry or bio-informatics” part of the procedure. In the case of NGS, the ComPerMed provided in 2016 guidelines with concise recommendations on all steps in the procedure in a generic, sequencing technology independent way. The guidelines are complementary to the ISO15189 norm for medical laboratories, support existing guidelines such as those of CAP, and are used as a reference by the national accreditation body Belac [7]. The NGS guidelines will not only facilitate the implementation of the required NGS-quality metrics for somatic variant detection in clinical laboratories, but also harmonize test validation, verification, clinical interpretation and reporting of variants. The guidelines serve in establishing in-house procedures and modalities for internal quality control (IQC) and provide the basis for establishing an external quality assessment system (EQA) to assure and maintain optimal test performance. The 2016 version of the NGS guidelines will be revised regularly and the ComPerMed intends to develop in future versions, some critical aspects in more detail including variant interpretation, bioinformatics handling and statistical model for the validation process.

### Action 3: Development of criteria for NGS use in oncology and hemato-oncology

**Fig. 3:**
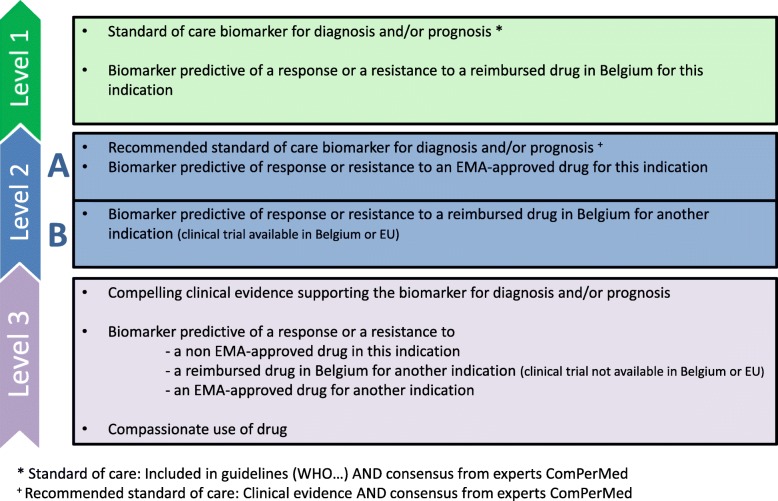
Definition of levels for diagnostic/prognostic or therapeutic biomarkers and molecular tests, according to the Belgian healthcare system

In this action, the scientific board of the ComPerMed has to determine for which indications NGS testing has an application in patient care. The process consists of defining 1°) the genes and/or gene regions to be analyzed by NGS for a given cancer based on literature and expert opinions, and 2°) the conditions for which this test should be performed for instance localized disease and/or metastatic disease. The final objective is to maintain only gene alterations with clinical utility in diagnosis and/or therapeutic choice and/or prognosis (patient outcome) in a specific cancer type. To accomplish this selection of genes, three different levels were defined to be able to categorize the genes according to standard vs. investigational therapeutic implication (Fig. 3) [8]. Levels linked to clinical guidelines, standard of care, expert’s opinion and approved drugs were determined from 1 to 3 with level 1 the highest level of therapeutic implication (standard of care and reimbursed drug). These levels are useful for classifying variants for NGS testing and molecular tests. Besides this, the agreement on algorithms displaying the work-up of a particular cancer type is forming the basis to define the specific conditions for NGS testing. The algorithms represent a sequential of molecular tests to be performed for a particular cancer, documented in addition with the clinical utility (diagnosis, prognosis or therapy), test level and a brief description of the molecular test. The purpose is to regularly revise the gene alterations to be analyzed and their level as well as to update the algorithms. This methodology will facilitate the implementation and standardization of NGS analysis in clinical practice as well as allow the development of reimbursement criteria for this test.

### Actions 4&5: Develop and organize a benchmarking trial and EQA program for NGS use in oncology and hemato-oncology

Application of NGS in clinical laboratories, as any other analysis, requires external quality assessment. In first instance, it was considered important to estimate the baseline performance of the clinical labs for their adequate manner of using NGS in their routine diagnostics within a benchmarking trial. The performance of NGS testing need to be evaluated on different aspects and sequentially with first, using samples from well-characterized genomic DNA from tumor cell lines to assess the quality of NGS analysis on determining the presence or absence of somatic variants and their allele frequencies. Secondly, extending these assessments to the pre-analytical conditions and to clinical interpretation, using FFPE or blood samples as start material and theoretical case studies for interpretation assessments. Consultation with all concerned parties is recommended for the development of the evaluation procedures. This action is performed together with a department of Sciensano, the department Quality of laboratories. In a final phase, it is essential to establish a national EQA program to monitor the quality of NGS testing in clinical practice and to propose corrective actions where needed.

### Action 6: Implement NGS registration, storage and data management

NGS analysis goes along with the generation of large amounts of data and the management of such information can represent an important added value for quality, outcome analysis and reimbursement reallocation as well as for clinical and public health research. The purpose of this action is to develop the technical platform for the central collection and storage of NGS data generated by licensed and accredited labs. In Belgium, this part of the NGS implementation is governed by the Healthdata.be services at Sciensano. The Healthdata.be service has a technical platform as part of the implementation of the e-Health 2013–2018 Action Plan of Belgium. Healthdata.be looks after the daily management and maintenance of this new technological tool, the objective of which is to bring all the data that is now stored in multiple health registers into a single Internet-based platform. Consequently, our platform contributes substantially to the provision of an infrastructural system dedicated to research and monitoring in Belgium. Irrespective of the topic, the data is recorded in Healthdata.be in a uniform and secure manner. This action requires the development of a data registration format and IT solutions for NGS data transfer to the Healthdata.be platform through the eHealth infrastructure. Also, an automatic link with the national Cancer Registry is being established. The ultimate goal is that NGS data are collected together with additional test results into a molecular registry improving accessibility of data for clinical research as well as facilitating evaluation and decision making for policy makers.

### Action 7: Provide NGS education and training

NGS is becoming more accessible for clinical labs but is also accompanied with various challenges. It is therefore necessary to provide education and training on the use of NGS. Defining the concrete needs in NGS education and training is done in concert with the healthcare sector, more specifically the College of Oncology, the College of Genetics, the Commission of Clinical Biology and the Commission of anatomic pathology. It is clear that different aspects can be covered including technical, legal and ethical aspects, as well as clinical applications and new evolutions towards third-generation long-range DNA sequencing, whole genome/exome sequencing, RNA sequencing, etc. The formats that can be used for post academic training and education are symposia, workshops, webinars and online courses. A first initiative was taken by the Molecular Pathology Working Group, where a two-day course was offered to residents in pathology. Participating on this course is now mandatory for all residents in pathology.

### Action 8: Informed consent, legal and ethical implications of NGS use in oncology and hemato-oncology molecular diagnostics

It is generally recognized that with the use of NGS technology in clinical practice, new legal and ethical points of attention emerge [9]. Besides generating useful clinical information, NGS testing can also reveal large number of data that may be considered disproportionate, although useful for research purposes and/or NGS can produce results that are not directly linked to the current cancer treatment. NGS data may in addition uncover relevant hereditary information, which leads to a whole new string of ethical issues. Another major issue with genetic/genomic information is privacy and data protection. A clear informed consent is considered essential when NGS techniques, especially WGS, or similar technologies are being applied for clinical use; this can prevent many misconceptions and avoid ethically problematic situations after testing. The aim of this action is to provide guidance on how the legal and ethical aspects related to the use of NGS can be managed. For this, it is essential to map the attitudes and information needs of cancer patients whom NGS testing is offered. Focus group studies and citizen labs will be installed to address preconceptions, doubts, expectations of the patients and citizens, the role of the healthcare professionals, access to information, data sharing, incidental findings, and comprehensiveness of NGS testing by patients and relevance for family members.

### Action 9: Pilot study ‘NGS use in routine diagnostics’

NGS implementation into the clinical routine diagnostics is a complex new intervention and will benefit from a transition period where the introduction of NGS testing itself is monitored. A real life pilot study can be put in place for this purpose through conventions of participating labs/hospitals with the reimbursement agency. At the end of this real life pilot study, an assessment will be made on the effectiveness of NGS in molecular diagnostics in oncology and hemato-oncology, including how this multi-testing approach can be positioned in the current reimbursement system, taking into account clinical utility, alternatives, therapeutic and societal need, quality and costs. The pilot phase will also allow to evaluate how a molecular data registration system can be best organized to allow documentation of test results and their use for later applications in quality control, output analysis and public health research.

### Action 10: Build on hospital networks for NGS use in oncology and hemato-oncology

Optimal use of NGS demands investments in infrastructure and technology, in trained laboratory staff and in IT; affecting not only the logistics of the laboratory but potentially also that of the whole hospital setting. An additional challenge for optimal NGS-implementation in a hospital-setting, aside from the logistics, includes providing adequate genomic oncology training to clinicians, pathologists and geneticists to facilitate the dissemination of genomic knowledge fit-for-purpose for patient management. In regions with multiple testing laboratories and hospitals, all with different practices on the implementation of these genomic tools in their daily practice and thus potentially with different impact on patient care, the establishment of a network-infrastructure between those hospitals will most probably be beneficial. Establishing infrastructures for these purposes requires a huge collaborative effort between all the stakeholders involved, namely oncologists, pathologists, organ-specific specialists, geneticists, patients and general practitioners, as well as healthcare system representatives from the Government in order to align medical genomic-driven practice with health care policy. It may therefore be considered beneficial for patient care to organize the testing, data analysis and interpretation within such a network infrastructure.

The development of regional NGS-network infrastructures should be supported by a thorough documentation of all quality-measures needed to establish optimal genomic-driven patient care. This includes documentation of the needed expertise of laboratory staff, documentation on how to ensure optimal interpretation of NGS-variants for daily practice, documentation on how to aim for optimal inclusion of patients in clinical trials, etc.….

The additional benefits of network-infrastructures for optimal NGS-use are 1°) to expand and link to existing national genomic medicine implementation initiatives; 2°) to develop new collaborative projects in diverse settings and populations within a region or between regions; 3°) to contribute in a more efficient way to evidence based use of genomic information for clinical care; 4°) to define, share and disseminate the best practices of genomic medicine implementation, diffusion and sustainability in diverse settings within a region and 5°) to more efficiently contribute to the development of a nationwide database containing specific genomic information in various histological types of cancer.

## Conclusions

Precision medicine has offered new opportunities in cancer care with biomarker testing playing a major contributive role in this process. As a consequence, targeted NGS is gradually introduced in molecular diagnostics in daily practice. It offers the advantage to obtain more information with less material, thereby replacing sequential biomarker testing. NGS raises a number of challenges concerning standardization, sample quality, use of different platforms, variant interpretation, reporting, data storage, reimbursement, and ethics. A roadmap outlining a number of actions to tackle these challenges is described in this paper. The actions include the development of guidelines and criteria for NGS by experts, quality assessments for NGS testing, installation of data registration, NGS education and training. Finally these actions need to be implemented in a real life pilot study that includes the formation of NGS networks. In Belgium, this roadmap is running and resulted in technical guidelines for NGS [7], in a document that is linked to the reimbursement of the NGS test, describing the genes and regions to be analyzed for specific cancers, and in a tool for data registration. Benchmarking studies to evaluate the quality of NGS testing in Belgian laboratories were performed, one for solid tumors and one for hematological myeloid cancers. NGS education and training is ongoing for pathologists and will be extended to other disciplines. In addition, cancer patients and citizens are involved in ethical debates and recommendations for policy makers and healthcare professionals will follow soon. The pilot study of 3 years will start in the near future and will facilitate the determination whether NGS and precision medicine improve patient healthcare and outcome. Furthermore, collecting the NGS-metrics for each Belgian NGS-reference center will enable us to benchmark the results of each laboratory with existing international benchmarks, like Cosmic or My Cancer Genome. Using this infrastructure will also enable us to centralize the collection of NGS data and allow us to participate in international data-gathering resources like GENIE. Finally, this program will enable the most optimal identification and integration of patients into clinical trials, as the percentage of variants encountered for a particular tumor type across the whole nation will be known.

In conclusion, this action plan can serve as a guide for similar initiatives by other countries to facilitate the implementation of NGS in clinical practice in oncology and hemato-oncology.

## Abbreviations

CAP: College of American Pathologists
ComPerMed: Commission of Personalized Medicine
ctDNA: Circulating tumor DNA
EQA: External quality assessments
FFPE: Formalin-fixed, paraffin-embedded
HTA: Health technology assessment
IQC: Internal quality control
NGS: Next-generation sequencing
NIHDI: National Institute for Health and Disability Insurance
